# Functional Intricacy and Symmetry of Long Non-Coding RNAs in Parasitic Infections

**DOI:** 10.3389/fcimb.2021.751523

**Published:** 2021-10-08

**Authors:** Joshua Seun Olajide, Bolatito Olopade, Jianping Cai

**Affiliations:** ^1^ State Key Laboratory of Veterinary Etiological Biology, Key Laboratory of Veterinary Parasitology of Gansu Province, Lanzhou Institute of Veterinary Research Chinese Academy of Agricultural Sciences, Lanzhou, China; ^2^ Centre for Distance Learning, Obafemi Awolowo University, Ile-Ife, Nigeria; ^3^ Jiangsu Co-Innovation Center for Prevention and Control of Animal Infectious Diseases and Zoonoses, Yangzhou, China; ^4^ Department of Medical Microbiology and Parasitology, College of Health Sciences, Obafemi Awolowo University, Ile-Ife, Nigeria

**Keywords:** long non-coding RNA, protozoa, helminth, transcripts, infection, parasite

## Abstract

RNAs are a class of molecules and the majority in eukaryotes are arbitrarily termed non- coding transcripts which are broadly classified as short and long non-coding RNAs. Recently, knowledge of the identification and functions of long non-coding RNAs have continued to accumulate and they are being recognized as important molecules that regulate parasite-host interface, parasite differentiation, host responses, and disease progression. Herein, we present and integrate the functions of host and parasite long non-coding RNAs during infections within the context of epigenetic re-programming and molecular crosstalk in the course of host-parasite interactions. Also, the modular range of parasite and host long non-coding RNAs in coordinated parasite developmental changes and host immune dynamic landscapes are discussed. We equally canvass the prospects of long non-coding RNAs in disease diagnosis and prognosis. Hindsight and suggestions are offered with the aim that it will bolster our understanding for future works on host and parasite long non-coding RNAs.

## Introduction

Genomic sequencing has continued to reveal an increasing number of transcripts termed non-coding RNAs (ncRNAs) due to the hypothesis that ncRNAs have no protein-coding potential. Meanwhile, advances in research are regularly giving evidence to show that some ncRNAs have protein-coding potentials (Matrajt, 2008; Vasconcelos et al., 2018; Fan et al., 2020), and the continuous identification and growing knowledge across large tracts of biological processes are beginning to uncover ncRNAs as important genomic transcripts (St.Laurent et al., 2015; Pawar et al., 2017). Eukaryotic ncRNAs are classified into short non-coding RNAs (sncRNAs) and long non-coding (lncRNAs) by the length of the nucleotide sequence as well as on the bases of their structures and functions (St.Laurent et al., 2015). As it is, lncRNAs form the largest group of RNAs with nucleotide lengths that span 200bp and100kb (Oliveira et al., 2018; Bensaoud et al., 2019). Essentially, unique features of lncRNAs include tissue-specific expression, poor sequence conservation (Liao et al., 2018), and low GC content (Petrella et al., 2015) with or without small open reading frames (Dhanoa et al., 2018; Mongelli et al., 2019). In addition, some lncRNAs are known to express functional micro-peptides that are no more than 100 amino acids (Rochet et al., 2019; Kim et al., 2020). The activities of lncRNAs are premised on their regulatory network as molecular decoys, scaffolds, guides, tethers to transcription factors, and sponges, especially in the cytoplasm. For a comprehensive description of lncRNA features as well as mechanisms of function and synthesis, reviews by Wang and Chang (2011); Beermann et al. (2016), and (Zhang et al., 2018) are important resources.

Moreover, lncRNAs may be functional during the development of organisms, cell proliferation, motility, inflammation, and gene regulation during host-pathogen interactions (Oliveira et al., 2018; Ren et al., 2018). These functional phenomena can occur through the binding of lncRNAs to RNAs and/or during transcription (Akay et al., 2019). Intrinsically, lncRNAs can form molecular complexes with DNA, mRNA, transcription factors, and heteronuclear proteins (Amit-Avraham et al., 2015; Menard et al., 2019) and could also affect mRNA stability or translation in the cytoplasm (Wang et al., 2014). lncRNAs can also influence gene regulation, chromatin modulation, and nuclear reconfiguration at various levels of biological processes (Li et al., 2020). Other functions of lncRNAs include imprinting, cell cycle regulation (Oliveira et al., 2018), and immune responses during infectious diseases (Rochet et al., 2019). Overall, however, functions of lncRNAs usually depend on cellular origin (Menard et al., 2018), species of organism, developmental stages, and correlated expression of genes (Vasconcelos et al., 2017; Vasconcelos et al., 2018).

Evidence has abounded to the point that lncRNAs are seen as significant supervisory molecules that intersperse regulatory mechanisms at various levels of physiological and pathological processes. Here, we discuss multiple layers of key regulatory functions of parasite and host lncRNAs in relation to infection of Apicomplexan (*Plasmodium falciparum*, *Cryptosporidium*, *Eimeria necatrix*, and *Toxoplasma gondii*), Kinetoplastida (*Leishmania* spp, *Tryoanosoma cruzi*), Parabasalia (*Trichomonas vaginalis*), and Helminth (*Schistosoma* spp, *Echinococcus granulossus* and *Toxocara canis*). This review seeks to expand and consolidate on the concept of RNAs in parasitism (Jarroux et al., 2017) by discussing the functions of lncRNAs in parasite developmental cycles, antigenic variation, epigenetic reprogramming, and parasite-host interactions. Equally, in respect of the hosts, predicted and functional immune regulatory functions of lncRNAs are discussed as well as their involvement in pathology and disease diagnosis. There are highlights on recent findings with the aim to unveil gaps in our understanding and to harness the growing knowledge for better insights into parasite biology and host responses.

## LncRNAs: Diversity, Transcription, and Localization

**Figure 1:**
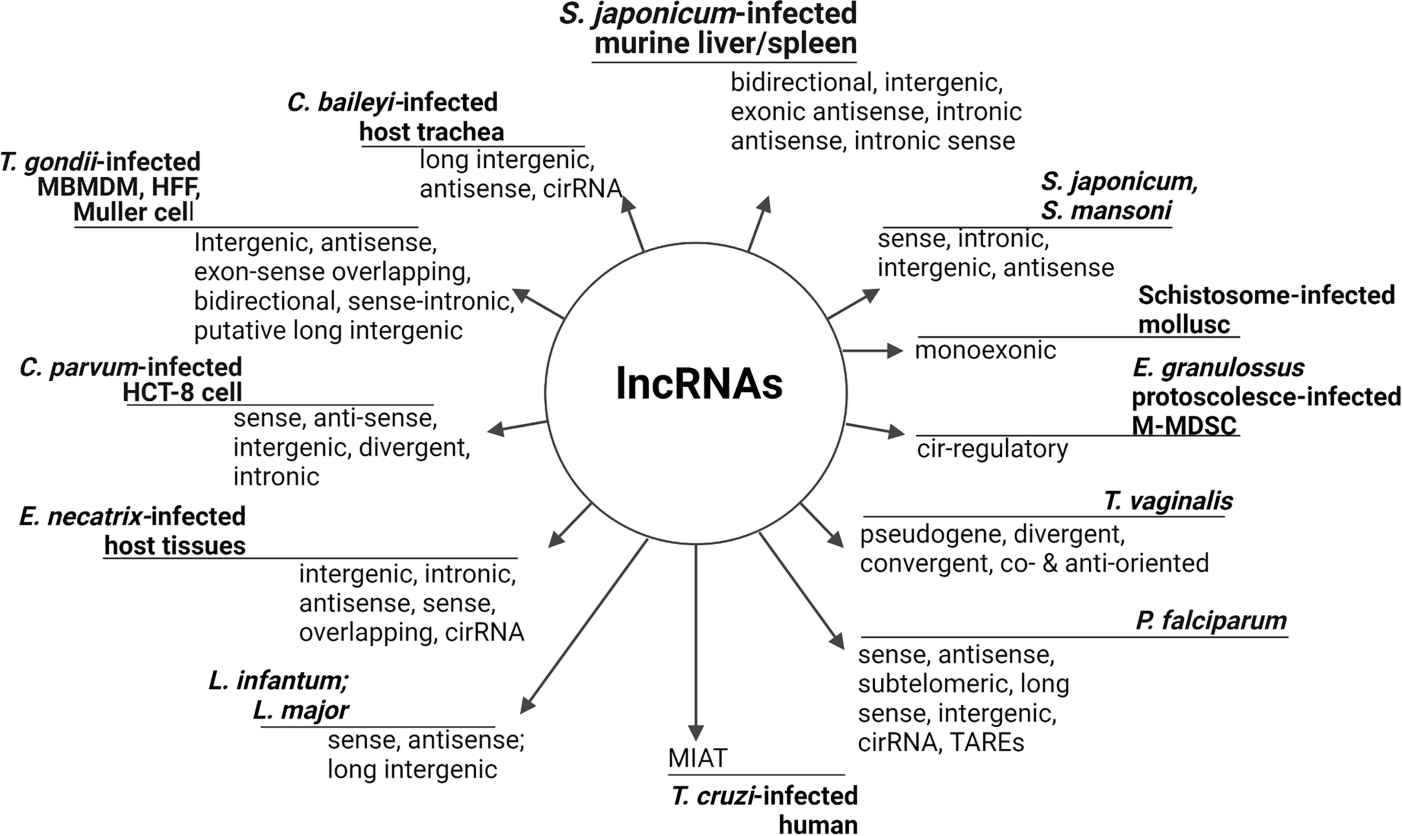
Identified lncRNAs in parasites and infected hosts/cells. LncRNAs are a diverse but distinctly defined RNA subset on the bases of their relative position to adjacent protein coding genes (Spurlock et al., 2016; Guidi et al., 2020), RNA resemblance, transcript sequence and structural conservation, biological function and biochemical pathways (St.Laurent et al., 2015), and genomic location (Vasconcelos et al., 2017). Long intergenic/intervening ncRNAs (lincRNAs), circular RNAs (circRNAs), and natural antisense transcript (NAT), (Kung et al., 2013) are common broad categories. Usually, lncRNAs are tissues/organ specific but parasite/host may have substantial tissue-overlapping lncRNAs that are associated with mRNAs as observed in *C. parvum*-infected cells. Long telomere-associated lncRNAs can be synchronously expressed with DNA replication while intronic lncRNA may be fragments of pre-mRNAs or expunged introns for degradation (Menard et al., 2021), whereas circRNAs are sponges for microRNA (Fan et al., 2020). LncRNA that are associated with protein coding genes are classified as lincRNAs, non-overlapping, intronic, antisense, bidirectional, sense, transcribed pseudogene (Loscalzo, 2014; Zhang and Cao, 2016), sense-overlapping, and long telomere-associated RNAs (Broadbent et al., 2011; Fan et al., 2020). Nonetheless, the levels of expression of lincRNAs are often lower compared to protein coding genes *ab initio* (Hassan et al., 2012). TARE, Telomere-Associated Repetitive Element; M-MDSCs, mice-monocytic myeloid-derived suppressor cells; HCT-8, human adenocarcinoma.

Identification of new lncRNAs is daily adding to the number of non-coding transcripts and sub-types in parasites and hosts (Kung et al., 2013) which, like in other eukaryotes, are categorized relative to nucleotide length, secondary structure, cellular localization (St.Laurent et al., 2015), and interaction with other nuclear elements (Dhanoa et al., 2018). The array of lncRNAs that have been reported in parasites and/or infected hosts cells are shown in **Figure 1** with their nominal classification and definitions. For further details on the structural classification of lncRNAs, reviews from Dhanoa et al. (2018); Zhang et al. (2018), and Marchese et al. (2017) are excellent resources. That said, lncRNAs are usually transcribed by the RNA polymerase II (Pol II)-dependent process which involves splicing, capping, and poly-adenylation (Bensaoud et al., 2019; Guidi et al., 2020) similarly to mRNA transcription (Marchese et al., 2017). Also, the transcription of lncRNAs is characteristically marked with sequence of initiation, elongation, and termination. However, unlike mRNA, lncRNA nucleotides have extensive translational stop codons (Aune and Spurlock, 2016), few exons, and lack an extended open reading frame (Pircher et al., 2014).

Taking clues from parasites, the schizont and ring stages of *P. falciparum* have heterogeneous lncRNAs that are transcribed from telomeric and sub-telomeric regions by RNA pol II (Sierra-miranda et al., 2012). Correspondingly, *L. infantum* promastigote and amastigote express lncRNAs that are transcribed by RNA pol II within sub-telomeric region and processed by trans-plicing and poly-adenylation (Dumas et al., 2006). However, *P. falciparum* antisense lncRNA is non-polyadenylated, independent of Pol II transcription, and its activation is sequence-specific in parasite late stages (Amit-Avraham et al., 2015). Remarkably, artificial *var* antisense lncRNAs have been transcribed using T7 RNA polymerase in *P. falciparum* (Jing et al., 2018) but the alternative pathway of lncRNAs transcription by RNA polymerase III (Mercer and Mattick, 2013) has not been reported in parasites. More studies are required, especially in non-apicomplexan protozoa and helminths, for empirical evidence on the possibility that lncRNAs may be contiguously transcribed differently in parasite stages, clade, or along non-coding repeat regions of a genome.

Across life domains, lncRNAs have shown rapid evolution, cellular specificity, and nuclear enrichment (Vasconcelos et al., 2017). In the nucleus, lncRNAs are involved in the regulation of nuclear organization (St.Laurent et al., 2015) as well as components of nuclear paraspeckles and matrixes, whereas cytoplasmic lncRNAs have been found in mitochondrion (Jarroux et al., 2017), and in association with ribosome and poly-ribosomes (Pircher et al., 2014). Growing evidence has also shown that lncRNAs can be selectively shed in extracellular milieu or enclosed in membranous vesicles (Dragomir et al., 2018).

*P. falciparum var* antisense lncRNA (Jing et al., 2018) and *L. major* promastigote lincRNAs (Misra et al., 2005) are localized to the nucleus, while *P. falciparum* schizont TARE6 lncRNA resides in a distict nuclear subcompartment without co-localization with the subtelomeric DNA clusters. This is an implication that the transcription of TARE6 lncRNA occurs momentarily after which it is organized into a new nuclear compartment (Sierra-miranda et al., 2012). Although *L. infantum* ‘intermediate’ sense and antisense lncRNA are oppositely transcribed, they are localized within the cytoplasm in a complex interaction with ribonucleo-protein (Dumas et al., 2006). Parasite lncRNAs can also be found in nucleolus (Sierra-miranda et al., 2012) or co-sediment with a specific sequence to form functional RNAs as observed in *T. vaginalis* genomic lncRNAs (Woehle et al., 2014). It may be valid, therefore, to state that lncRNA localization and transcription can occur differently with respect to parasite species, stage of development, and genomic structure. In comparison with other eukaryotes, ribosome-associated lncRNA (Pircher et al., 2014) has not been reported in parasite, but if found, it may likely impact substantial gene expression and translation in response to environmental changes.

## Roles of LNcRNAs in Parasite Development

**Figure 2:**
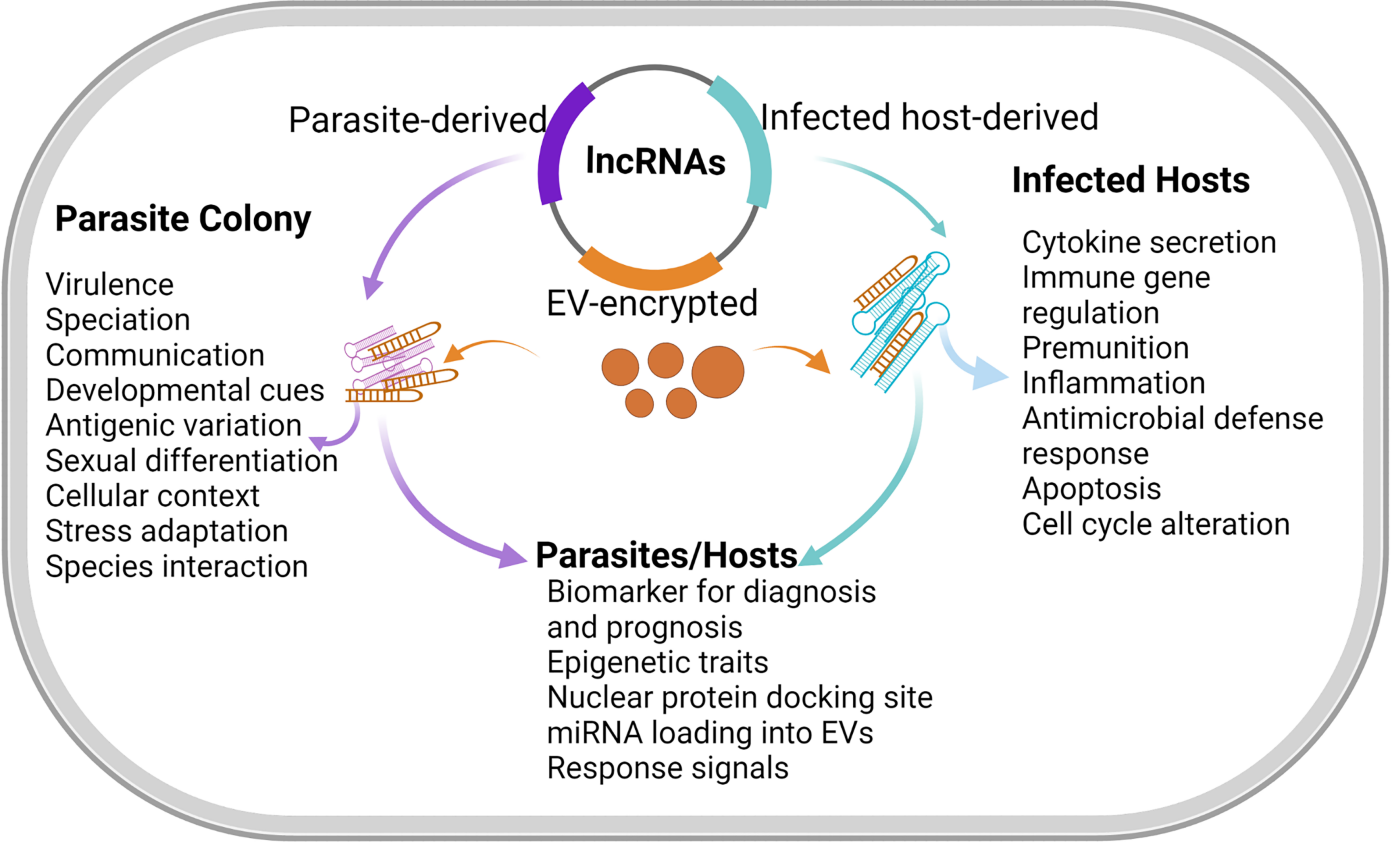
Functions of lncRNA in hosts and parasites. LncRNA expressions are usually induced during genetic and physiological stress (Atkinson et al., 2018). The functionalities of lncRNAs are inherently numerous including molecular signals, spatio-temporal transcription to integrate developmental cues, cellular context, and responses to diverse stimuli. LncRNAs that integrate contextual and environmental cues can be found during developmental stress and apoptosis. LncRNAs may similarly act as regulatory knobs in many transcriptional pathways (Wang and Chang, 2011). However, lncRNA may interact with the transcription factors to limit the expression of pro-apoptotic genes and thus enables cell-cycle arrest, which is suggestive of extensive roles of lncRNAs in cell development. Meanwhile, the exchange of lncRNAs through membranous vesicles circulating during infections could facilitate additional functions of lncRNAs in disease diagnosis and prognosis.

Parasitic organisms have a multi-stage life history along which organismal complexity increases and the need for requisite adaptation in specific host (Kafsack et al., 2014). The abundance of lncRNA have some level of correlation with parasite development, cellular differentiation, and identity (Kim et al., 2020). First, lncRNAs are seen as key regulators of sexual development in protozoa and helminths. In a study of schistosome population, there were differentially regulated lncRNAs in paired (adult male and female), unpaired (female only), and ovaries of *S. mansoni*. This in effect demonstrated the possibility that lncRNAs could guide the process of sexual recognition, maturation, and reproduction in sexually dimorphic helminths (Amaral et al., 2020). In protozoa, lncRNA has also been associated with parasite sexual differentiation as long non-coding *gdv1* antisense RNA negatively regulate *P. falciparum* gametocyte sexual commitment *via* gametocyte development protein 1 (GDV1) by interfering with transcription, stability, or translation of *gdv1* mRNA (Filarsky et al., 2018). Further work is required to find out the extent to which lncRNA could synergize parasite sexual differentiation or gametocyte sorting (**Figure 2**). This may be an important process that can be explored to halt parasite development and disease progression.

Furthermore, the expression of lncRNA might differ across developmental stages of a parasite (Michaeli et al., 2012) or it could be developmentally regulated. For example, *S. mansoni* sporocysts, adult male and female populations, and male-only adults express common and unique lncRNAs during development (Kim et al., 2020). Consequently, up-regulation of some lincRNAs in adult *S. mansoni* in comparison with schistosomula (free-living larvae) suggests lncRNAs might play crucial roles in the rapid transition and adaptation of adult *S. mansoni* to a parasitic mode of life in mammalian host (Kim et al., 2020; **Figure 2**). Also, bioinformatics analysis has shown that specific telomere-associated lncRNAs may play significant roles during the development of *P. falciparum* schizont to ring stage (Broadbent et al., 2011).

The expression and function of lncRNAs may traverse several developmental stages or be limited to a specific stage of the development in response to various environmental, adaptational, or biochemical cues. Along the *P. falciparum* life cycle, some lncRNAs in the schizont stage were missing in the trophozoite, indicating that the entire activation of these lncRNAs occured in the schizont and their disappearance in trophozoite may be linked to translational process (Sierra-miranda et al., 2012). As such, the predominance of some lncRNAs across developmental stages may have important roles in parasite developmental transitions or stage-specific roles. Also, the iterative rounds of parasite development in different (living) environments are likely to contribute to alterations, regulation, composition, and stability of lncRNA. For instance, *L. infantum* amastigote-specific regulatory expression of intermediate ncRNAs failed in episomal expression vector as well as in promastigotes (Dumas et al., 2006).

It is also likely that, as development progresses, organisms acquire more lncRNA genes and transcripts to guide developmental complexity (Aune and Spurlock, 2016). Unlike sense transcript, *P. falciparum* antisense lncRNA showed negligible expression in *Anopheles gambiae* during sporogonic phase but was highly expressed in gametocytes and during ring stage (Gómez-díaz et al., 2017). Further, antisense lncRNAs were detectable from late ring-stage to intra-erythrocytic stage of *P. falciparum* (Amit-Avraham et al., 2015) and, during *P. falciparum* developmental progression, the expression pattern of lncRNA-TARE-4L coincides with DNA replication and parasite schizogony (Broadbent et al., 2011). Among multi-cellular parasites exemplified by schistosomes, up-regulation of schistosomula lincRNA may well point to it as a regulator for worm body re-modeling and rapid adaptation (Vasconcelos et al., 2017). As development continues, some lncRNAs could become relatively stable, being under strict control for stage-specific expression or function (Wei et al., 2019). There can also be stably silent lncRNAs during parasite development in host (Rochet et al., 2019), such as the quiescent long non-coding transcripts that later assumed regulatory function when *S. mansoni* sporocysts were exposed to different environments (Kim et al., 2020) (**Figure 2**).

There are reports of similar and/or different expressions of lncRNA in parasite strains, stages, and species (Rochet et al., 2019; Kim et al., 2020). Among *T. gondi* strains, significant lncRNAs were found to be differentially expressed or modulated (Rochet et al., 2019). Such relative lncRNA expressions are extant intra/inter-species features (Leitão et al., 2020) that could be useful bio-systematic tools to define species relatedness as reported among *S. mansoni*, *S. haematobium*, and *S. japonicum* (Liao et al., 2018). In this way, lncRNAs can delineate related species/strains by considering the aptness of genomic lncRNA transcription, differential abundance, and activity of lncRNA promoter that activate or inactivate the same gene or corresponding gene (Jing et al., 2018) to give a characteristic lncRNA expression in parasite species. In essence, differences in activation of lncRNA gene promoter at the same locus could translate to different expression of lncRNAs in different species or strain. But given the variations in the level of parasite genomic compactness and/or species complexity, different parasites may employ varying measures of gene induction for lncRNA activation, and the factors that initiate gene induction are also important.

During *T. gondi* tachyzoite development in host, there were time-dependent up-regulation and down-regulation of lncRNAs all through the active replication and tachyzoite egress in human retinal Müller cells (Rochet et al., 2019). Similarly, myocardial infarction–associated long non-coding transcript (MIAT) was found to be differentially higher among human males than females with chronic cardiomyopathy due to chagas disease (Frade et al., 2016). Thus, lncRNAs could mediate parasite transition in the hosts by hijacking specific host process of cell differentiation, homeostasis, and gene expressions (**Figure 2**) but the underlining mechanism by which parasites preferentially up-regulate lncRNA expression in certain host sex as well as parasite replication in such hosts are still unclear. In addition, during parasite developmental changes, lncRNAs may unlock specific genes for adaptable changes, differentiation, gene silencing, and expression in *Plasmodium*, and possibly other multi-cellular parasites. More studies on lncRNA expression patterns between parasite life stages within and outside the host would increase our understanding of parasite propagation, transcriptomic regulation of sexual differentiation, and host permissiveness.

## Parasite Epigenetic Regulations by LNcRNAs

The uniqueness of lncRNAs relies on their ability to bind proteins and nucleic acids through which their activities are reinforced (**Table 1**). By this molecular magnate, lncRNAs may mediate epigenetic events (i.e. chromatin modifications) to activate transcriptional reactions (Vasconcelos et al., 2017). Reports from studies have identified lncRNAs as vital molecules in epigenetic regulation/modulation (Amit-Avraham et al., 2015) by integrating feedback processes from intracellular trafficking and chromosomal transformation (Bensaoud et al., 2019; Broadbent et al., 2011) during transcription or post-transcription (Frade et al., 2016; Fan et al., 2020). Specifically, lncRNAs are an emerging paradigm in epigenetic remodeling of malaria parasite (Broadbent et al., 2011) that culminated in substantial expression of virulence genes (Sierra-miranda et al., 2012) involving histone modifications and nuclear re-organization in the parasite blood stages. Also, the expression of antisense lncRNA resulted in the activation of *P. falciparum* mRNA of an active gene (Gómez-díaz et al., 2017). It is suggestive, therefore, that lncRNAs, by conformational rearrangement, can influence epigenetic traits in parasite but the extent, aside gene activation, is not known. It is likely that such swift, re-programmed gene activation, or its intended phenotype, would influence successful establishment of parasite in host or show deleterious effects in the parasite.

**Table 1:** Specific function of lncRNAs in host and parasite.

Parasite Spp	Parasite- or Host-derived	lncRNA	Predicted/Potential Target(s)	Function	Reference
**Protozoa**					
*T. gondii*	Host fibroblast fore skin	NONSHAT022487	UNC93B1 immune related genes	mediates secretion of IL-12, TNF-α, IL-1β and IFN-γ by negative expression of UNC93B1	Liu et al., 2018a
*T. gondii*	Mouse BMDM	Csf1-lnc and Socs2-lnc	kinase ROP16	Up-regulation of lncRNAs Csf1-lnc and Socs2-lnc,	Menard et al., 2018
*C. parvum*	Murine IEC4.1	NR_045064	Csf2, Nos2, and Cxcl2	promote epithelial antimicrobial defense	Strauss-soukup and Chen, 2019
*C. parvum*	HCT-8 cell line	sense, antisense, intergenic, divergent and intronic	hedgehog, Wnt signaling pathways, tight junction	**^p^** maintenance of intestinal epithelium integrity	Liu et al., 2018b
IId subtype					
*T. gondi* tachyzoite	Human Retinal Müller Cells	NeST, MEG3, MIR17HG, lnc-SGK	Th1 and Th17	**^p^** immune responses	Rochet et al., 2019
*T.gondii* RH	Mice BMDM	mir17hg	host gene for mir17 microRNA cluster	**^p^** apoptosis	Menard et al., 2021
*C. baileyi*	Host trachea tissue	lncRNAs, cirRNA	?	**^P^** cytokine-cytokine interaction cell cycle, IgA production metabolism, tight junction	Ren et al., 2018
*E. necatrix*	Chicken intestine	NONGGAT004163.2, TCONS_00018115, NONGGAT001393.2	ring finger protein 152 type I interferon rec- eptor subunit 1	**^p^** apoptosis host defense against foreign pathogens	Fan et al., 2020
*P. falciparum* trophozoite schizont merozoite	parasite	Long antisense ncRNA	var genes PFF0845c PFD1005c	gene regulation	Epp et al., 2009
*P. falciparum*	parasiteblood stage	lncRNA-TARE	parasite DNA replication	parasite blood stage development	Broadbent et al., 2011
*P. falciparum*	parasite asexual blood stage	*var* antisense lncRNA	Parasite var genes	induce var gene transcription activation, and promoter activity	Jing et al., 2018
*P. falciparum*	Parasite red blood cell stage	lncRNAs	?	**^p^** Host interaction, proteolysis, cell adhesion, locomotion, pathogenesis, metabolism	Liao et al., 2014
T. cruzi	heart ventricular tissue	MIAT	?	chronic cardiomyopathy due to chagas disease	Frade et al., 2016
**Helminths**					
*E. granulosus*	Mice splenic M-MDSCs	NONMMUT021591	cis-regulation of retin- oblastoma gene, Rb1	**^p^** abnormal M-MDSCs differentiation	Yu et al., 2018
*Toxocara canis*	Dog lungs	XLOC_030813, XLOC_510697, XLOC_237221	Regulation of ubqln1, inhibit sox4 expression IL-21 gene localization	**^p^** immune- or inflammation- related function	Zheng et al., 2021
*S. mansoni*	adult worm	putative lncRNAs	sexual dimorphism and drug sensitivity	**^p^** metabolism, transport biosynthesis, nucleotide binding drug sensitivity, catalytic activity	Oliveira et al., 2018
*S. mansoni*	cercariae schistosomula	SmLincRNAs	parasite transition sex differentiation	**^p^** parasite development	Vasconcelos et al., 2017
*S. japonicum*	Mice liver, spleen	NONMMUT014792.2, NONMMUT061096.2, NONMMUT057813.2, NONMMUT057813.2	TGFβ-1, JAK3, STAT1 regulation chemokine C motif receptor 1, VCAM1	**^p^** liver pathogenesis	Xia et al., 2020

M-MDSCs, mice-monocytic myeloid-derived suppressor cells; TARE, telomere-associated repetitive element transcripts; VCAM1, vascular cell adhesion molecule 1; XCR1, chemokine C motif receptor 1; ^p^prediction by functional annotation/correlation network analysis.

In response to *C. parvum* infection, *Nos2* and *Csf2* were transcriptionally controlled by NR_045064 in conjunction with methylation of histone and co-activation of other genes whose translational products regulate transcription and mediate disease development (Strauss-soukup and Chen, 2019; **Table 1**). For blood stage *P. falciparum*, lncRNA-TARE could edge chromatin synthesizing factors to modulate specific epigenetic process of adjoining sub-telomeres (Broadbent et al., 2011). Likewise, lncRNAs could substitute RNA genes and, in the process, coordinate genetic regulatory outputs (Rinn and Chang, 2012) with extremely diverse and substantial functional plasticity that rely on lncRNA nucleotide bases, structural conformity, and molecular interactions (Marchese et al., 2017). However, in this respect, antisense RNAs can also silent epigenetic mechanism and catalyze the formation of heterochromatin in *P. falciparum* (Broadbent et al., 2015).

Since epigenetic marks are histone-bound, H3K9 (Histone 3, lysine 9) trimethylation mark has been proposed as the basis for *P. falciparum var* gene repression outside coding region which was either greatly acetylated while active or massively trimethylated when silent (Lopez-Rubio et al., 2007). The genetic drive for lncRNA acetylation in parasite requires further evidence as it could either influence gene activation or confer epigenetic methylation during the formation of heterochromatin. An example of direct transcriptional activator for epigenetic mark is *P. plasmodium* DNA-binding protein, PfAP2-G, which is crucial for gametocyte formation. The *pfap2-g* locus shows epigenetic silencing of multi-gene families especially by H3K9me3 histone modulation that is typical of repressing chromatin structures in a reversible formation (Kafsack et al., 2014). This process of *pfap2-g*-mediated suppression of epigenetic regulation in *P. plasmodium* may likely involve lncRNA, but this assumption needs to be substantiated.

Another emerging mechanism, involving epigenetics alongside lncRNA regulations, implicates drug treatment or exogenous triggers that are capable of orchestrating changes in chromatin conformations and translational processes. Such treatment has been shown to impart higher growth rate in *Plasmodium* parasite expressing episomal antisense lncRNAs than un-transfected or mock-plasmid transfected parasites (Amit-Avraham et al., 2015). Similarly, lncRNAs were differentially regulated in 5−azacytidine-treeated *S. mansoni* populations, suggesting epigenetic regulation by drugs (Amaral et al., 2020), but the mechanisms presupposing these actions are not known. Nevertheless, studies on differences in lncRNAs expression and function could help to distinguish corresponding epigenetic changes in parasite and the heralding epigenetic factors. It would be important to find the degree to which external factors modulate the entire parasite transcriptome as well as lncRNA transcription/activation to render epigenetic traits (**Table 1**). Moreover, it is yet unknown if lncRNA-mediated epigenetic landscapes are reversible.

## LNcRNAs as Chaperons for Antigenic Variation and Virulence

Antigenic variation is a complex process orchestrated by epigenetic elements and controlled by different factors, but not DNA rearrangement (Lopez-Rubio et al., 2007). Antigenic or phenotypic variation of surface-exposed antigens allows parasites to induce chronic and recurrent infections (Prucca et al., 2008) by switching the expression pattern to sustain infections. In contrast, virulence, at the least, is attributed to the ability of parasite to escape host defense systems by consistently varying antigenic conformations (Amit-Avraham et al., 2015; **Table 1**). In both cases, depending on parasite species, different mechanisms have been proposed and regulation of genes by lncRNAs is adding the molecular strata of parasite antigenic re-combination, immune escape, or virulence. The poor conservation of lncRNA across species (Rochet et al., 2019) is of great application in this regard, though the exact roles of lncRNAs as chaperons for virulence and antigenic variation have not been completely charted in many parasites.

The function of lncRNAs in antigenic variation is partly connected with their tendency to flank protein coding genes and thus transcriptionally influence rapid adaptation of parasites to diverse environments by consistently changing the surface antigens (Oliveira et al., 2018). In addition, multi-gene families located in the vicinity of sub-telomeres are pertinent to parasite antigenic variation (Matrajt, 2008). In malaria parasite, *var* genes, a cluster of multicopy gene, have been demonstrated with var-luciferase transgenic *P. falciparum* to be activated by steady transcriptional overexpression of specific antisense lncRNA (Amit-Avraham et al., 2015).

Also, the transcription of antisense lncRNA could synchronize with the activation of its analogous *var* gene and promoter. In this case, *var* genes encode *P. falciparum* erythrocyte membrane protein 1, a virulence factor, that was subjected to adaptable switches for variant antigen expression after the activation of antisense lncRNA (Jing et al., 2018). Consequently, the expression of *var* genes correlates with the transcription of corresponding antisense lncRNA after *P. falciparum* invasion, which accordingly points to the fact that lncRNA may influence switching of *var* genes and subsequent translation of antigenic proteins on *P. falciparum*-infected RBCs (Jiang et al., 2013). Also, var antisense lncRNA exerts an activatory function during the transcription of *var* gene to the point that the earlier activated and nascent *var* gene mRNAs co-exist in the same parasite (Jing et al., 2018) but sequential translational processes of both mRNAs were not reported.

Multiple *var* genes encode diverse antigenic proteins in *Plasmodium*, *Trypanosomes*, and *Giardia*. Some of these *var* genes may be expressed or remain silent simultaneously by mutually exclusive gene expression through DNA rearrangement and modification (Jing et al., 2018; Pays et al., 2004). Equally, genes that regulate parasite virulence (Cross, 1996) may overlay lncRNAs that *cis-* or *trans-*regulate gene switching for antigenic variation and, in such case, lncRNA could concurrently regulate antigenic variation and virulence. Conversely, exogenous antisense lncRNA could prompt the transcription of dormant *var* gene in *Plasmodium* to induce ‘competitive transcription’ which decreases the transcriptional dominance of already activated *var* gene. This dual transcription could modulate switching of *var* genes to enhance antigenic change (Jing et al., 2018). Also, the use of peptide nucleic acids as complement interference on antisense lncRNAs stimulated the suppression of an active gene, obliterated epigenetic memory, and induced the transcription and translation of inactive genes (Amit-Avraham et al., 2015). It is imperative, therefore, to determine the extent to which the nascent or co-expressed active genes confer virulence, antigenicity, drug susceptibility, or immune escape on parasites after stimulation by lncRNAs.

The surface expression of antigenic variation can in some cases be due to changes in heterochromatin structures or lack of expression by certain genes. *P. falciparum* variant-silencing SET gene (*PfSETvs*) knock-out enhanced the expression of antigenic proteins by histone H3 lysine 36 trimethylation (H3K36me3) of *var* genes. Jiang et al. further revealed that *var* gene in wild type *P. falciparum* had low levels of H3K36me3 and that silent *var* genes displayed high H3K36me3 methylation at the same exonic region to indicate a positive correlation between *PfSETvs*-dependent methylation and *var* lncRNA silencing (Jiang et al., 2013). Given lncRNA polymorphic sequence and binding tendencies to DNA, RNA, and proteins, the suggestion that antisense lncRNAs can activate the expression of *var* and non-*var* gene promoters is possible (Jiang et al., 2013) but lncRNA potential biding domains, preference, and affinity for nucleic acids and protein need further investigation with respect to parasite antigenic switches.

Furthermore, conservation of specific lncRNA expression across virulent and highly virulent *T. vaginalis* strains (Woehle et al., 2014) have been reported to demarcate the degree of inferred pathology in the host cell (**Figure 1**). Differentially abundant and regulated lncRNAs particular to *T. gondi* high-virulent strain have been observed in mice bone marrow-derived macrophage (BMDM) when infected with *T. gondi* high- and low-virulent strains (Menard et al., 2018) in which the virulent *T. gondi* strain was able to trigger higher expression of infection-related long noncoding transcripts than the less virulent strain (Menard et al., 2021). Additionally, lncRNA expressions during *S. japonicum* infection in mice may not be unconnected with parasite pathogenesis or virulence pathways (Xia et al., 2020)(**Figure 2**). Nevertheless, the expression of lncRNA during parasite infection may be of host particular responses, among other things, and as such, it could overtly depend on host infected tissues and species. The extent of lncRNA expression in host in response to parasite virulence must therefore be described in line with host genetics and transcriptomic signatures (e.g. outlier and allele-specific expressions) rather than parasite virulence *sensus stricto*.

## Re-Definition of Host-Parasite Interactions

LncRNAs are being reported as functional molecules in host-pathogen interactions (Liu et al., 2018b). During such dialogue, host and parasite lncRNA genes are concomitantly expressed at some point during the course of infection (Broadbent et al., 2011). However, host-derived lncRNA expressions and regulatory roles may change consistently during pathophysiological conditions (Rochet et al., 2019) so much that disease-associated and pathogen-induced lncRNAs become more abundant (Mongelli et al., 2019). These parasite-induced host lncRNAs (**Figure 1**) and corresponding genes could either be up- or down- modulated (Strauss-soukup and Chen, 2019) and the expression levels could vary with host cell type, parasite species/strains, and duration of infection.

NR_045064 was found up-regulated and finely controlled in *C. parvum-*infected mice intestinal epithelial cells (IECs, **Table 1**) as well as in the brain, heart, and lungs (Strauss-soukup and Chen, 2019) to signify that the parasite may co-opt the expression of specific host lncRNA in different tissues. On the contrary, during *T. vaginalis* infection in human and mice, the parasite lncRNA population had a considerable percentage of the total transcripts (Woehle et al., 2014). It is, then, not clear if overbearing of parasite lncRNAs, in host, is a sign of established infection or if identification of the same lncRNA in different tissues marks hyper-expression of such lncRNA in parasitic disease or its specificity to the parasite infection, knowing that lncRNAs are tissue-specific.

Apart from lncRNA specific tissue expression in pathophysiology, they are also vital indicators for cellular stress and senescence. Sensitivity to stress in host by *S. mansoni* is attributable to the expression of Sm-lncRNA5 and Sm-lncRNA12 which are in turn associated with ubiquitination, proteasome regulation, and cellular degradation (Oliveira et al., 2018). Also, secretion of *T. gondi* rhoptry kinase 16 regulates several putative host lncRNAs (Menard et al., 2018) during host cell invasion (**Table 1**). The majority of host cell lncRNAs were also down-regulated after infection of *T. gondii* with simultaneous synchronization of tachyzoite egress and cell death (Rochet et al., 2019). Incidentally, lncRNAs have been associated with parasite pathogenesis and apoptosis (**Figure 2**). It is likely that other forms of cell death (such as necroptosis and pyroptosis) that have not attracted research interest in parasitic infections may have some underlying mechanisms that involve lncRNAs.

**Figure 3:**
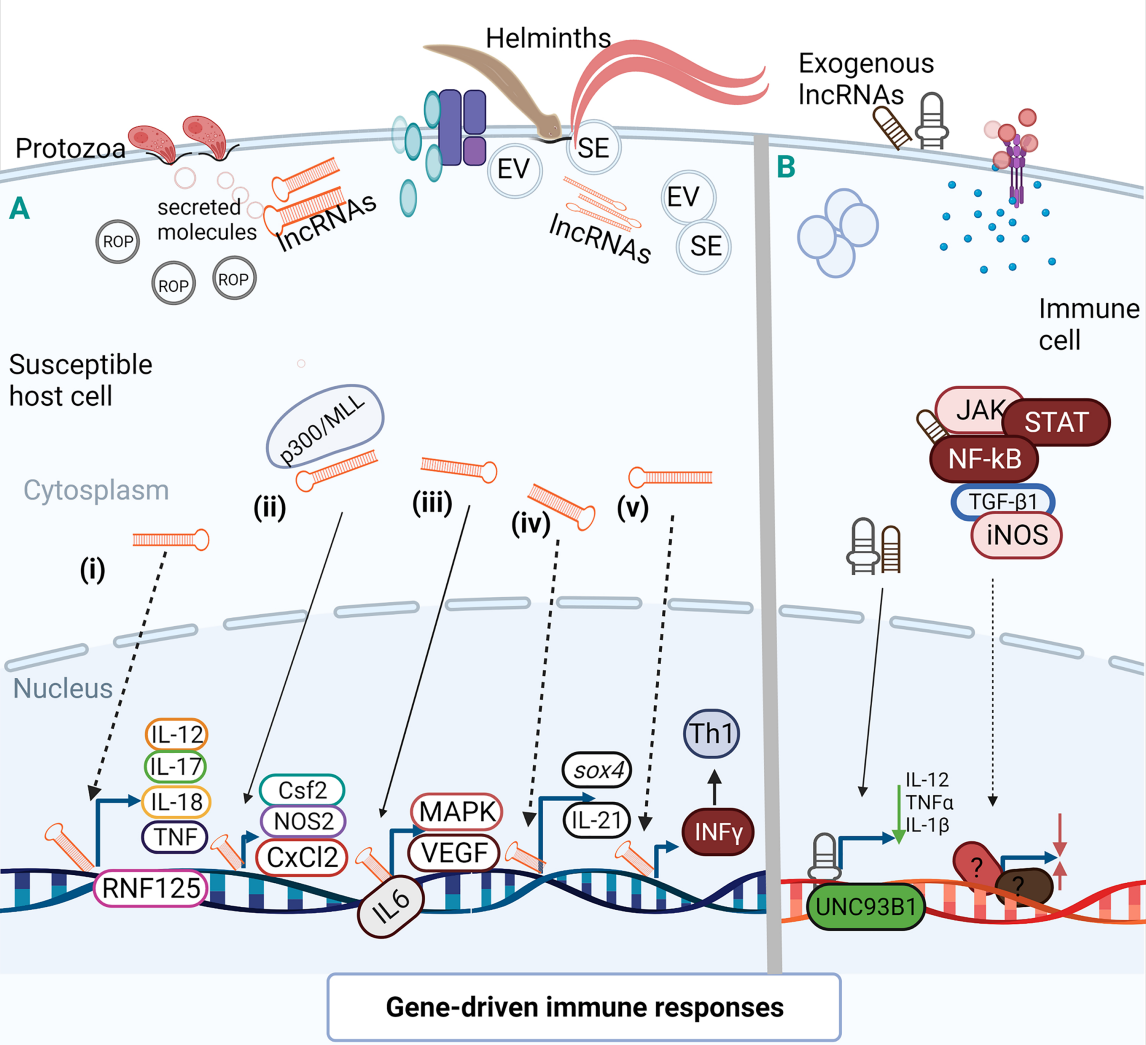
Activation and suppression of immune genes by lncRNAs. LncRNAs can act allosterically on gene regulatory domains and modify structural conformations to activate or suppress the function of related domains (Mercer and Mattick, 2013) for gene activation or chromatin conformation. **A(i)**. During HCT-8 infection with *C parvum*, specific lncRNA could target RNF125 by regulating the expression of RNF125 and possibly cause the expression of several inflammatory cytokines. **A(ii)**. Epigenetic histone modification by lncRNA-mediated transcription of host defense genes as observed during *in vitro* infection of *C parvum* with IECs. This chromatin remodeling of lncRNA *via* complex interaction with WDR5/MLL-p300 mediated the transcription of host cell defense genes where lncRNA over-expression enhanced expression of *Csf2*, *Nos2*, and *Cxcl2*. **A(iii)**. In M-MDSC, IL-6 may act as a transcription factor for some lncRNAs during *E granulosus* infection to prime MAPK and vascular endothelial growth factors (VEGF). **A(iv)**. Up-regulation of lncRNA leads to inhibited expression of *sox4* which in turn cause reduction in the healing of parasite-induced wounds to allow parasite migration. Also, the binding of lncRNA to a specific locus may downplay the transcription of IL-21 in a time dependent manner for persistent infection. **A(v)**. In human retinal Müller cell, NeST may induce IFN-γ transcription to enhance Th1 response during infection with *T. gondii*. **(B)** After *T. gondii* infection of human macrophage, lncRNA suppressed the expression of UNC93B1, an immune molecule, and decreased the secretion of inflammatory cytokines. During differentiation of human dendritic cells, lncRNAs may mediate activation of transcription signal transducer and activator of transcription 3 (STAT3) to promote its phosphorylation on tyrosine-705 by preventing STAT 3 binding or de-phosphorylation of SHP1 (Wang et al., 2014) but the molecular interaction of this manner has not been identified in parasite-infected cells. (Broken arrow; predictive function, unbroken arrow; validated function).

Functional transfer of lncRNAs could be mediated by extracellular vesicles (EVs) as communication channels that vehiculate the transfer of ncRNAs during host-parasite interactions. There has been demonstration of inter-communication between *Plasmodium* and host cell that was facilitated by ncRNAs (Leitão et al., 2020). It is expected that selected lncRNAs in extracellular vesicles (EV) or secretome (SE) are involved in host-parasite interactions (Olajide and Cai, 2020; Moreno et al., 2021) (**Figure 3**). However, there is yet to be an explicit definition and identification of parasite lncRNAs in parasite-derived EVs and their possible inter-reactions with those of host origin. Also, helminths are known to possess an attachment organ which can equally serve as channels for secretomes (Moreno et al., 2021). It would benefit our understanding to know what sorts of lncRNAs are involved in such SEs during interaction with the host and, possibly, if molecular sorting/switching is equally possible to avoid being sloughed off or the death of the host cells (**Figure 3**).

## Activation of Host-Immune Genes

From experimental observations and computational arrays, lncRNAs are involved in innate and adaptive immune systems (Liu et al., 2018a; Menard et al., 2021) as regulatory nodes for activation and amplification of immune signals, transcriptional factors (Wang and Chang, 2011), as well as co-regulator of infection- and immune-related genes (Menard et al., 2018)(**Figure 3**). In these processes, lncRNAs may integrate pro-inflammatory and anti-inflammatory responses, immune cell differentiation, and cytokine secretion or inhibition (Menard et al., 2018) (**Figure 3**).

The functional induction of specific lncRNA has been shown to orchestrate the transcriptional regulation of IEC defense genes during infection with *C. parvum* (**Table 1**) (**Figure 3**). Similarly, the induction of NR_045064 enforced the transcriptional regulation of host cell defense genes after infection with *C. parvum* (Strauss-soukup and Chen, 2019) (**Table 1**) just as over-expression of a lncRNA negatively regulated the expression pattern of UNC93B1 and secretion of pro-inflammatory cytokines in *T. gondii*-infected cells (Liu et al., 2018a; **Figure 3**). There was also computational prediction that XLOC_001265 could be involved in pro-inflammatory reaction that is dependent on the regulation of ring finger protein (RNF) 125 in response to *C. parvum* infection (Liu et al., 2018b).

Co-expression network and correlation analysis have revealed mutual expression of lncRNAs and immune genes as well as protein during infection with *T. gondii* (Liu et al., 2018a; **Table 1**). In this manner, the differentially regulated lncRNAs during *E. necatrix* infection might down-regulate host defense genes through recruitment of toll-like receptor and/or induce phosphorylation to activate inflammatory reactions (Fan et al., 2020). The *in silico* concomitant reduction of IL-21 and XLOC_237221 in dogs infected with *T. canis* requires functional analysis to substantiate humoral immune response and production of antibodies (Zheng et al., 2021) (**Figure 3**). *T*. *gondii* and *T. canis* are respectively entrenched and emerging zoonotic species while *E. necatrix* is of great veterinary importance. Functional analysis of lncRNAs in relation to host defense against these parasites would reveal a new dimension of immunity and control.

Again, bioinformatics analysis has indicated an association of lncRNAs with macrophage differentiation, cytokine-receptor interaction, JAK-STAT, and p53 signaling pathways during *T. gondii* infection (Menard et al., 2018) (**Figure 3**). MAPK has been implicated in some parasitic infections and now lncRNAs are being seen as regulator of inflammatory process in mammalian leukocytes (Agliano et al., 2019). Also, NF-κB activation is reminiscent of lncRNA genes expression as essential components of transcriptional feedback to infection (**Table 1**). However, the potential transcriptional and translational components of NF-κB-mediated sequence need further elucidation (Strauss-soukup and Chen, 2019). Likewise, differentially expressed lncRNAs by different *T. gondii* strains may have cardinal roles in MyD88-dependent protection in mice (Menard et al., 2021). It is conceivable that host cells may express lncRNA to undermine pathogens and pathogens, as well, may also utilize host lncRNAs to foil induction of host gene expression (Loscalzo, 2014; **Figure 3**) but these, too, require further clarification.

## Diagnostic and Therapeutic Prospects

Functional and genetic evidence are increasing in support of lncRNA anti-parasitic activity and involvement in disease diagnosis. It was earlier reported that increased expression of MIAT in chagas disease was associated with endothelial dysfunction in chronic cardiomyopathy, and it had a positive predictive value that signified putative correlation with *T. cruzi* parasitemia in mice (Frade et al., 2016). In addition, differential MIAT gene expressions in *T. cruzi*-infected subjects with chronic cardiomyopathy and non-infected subjects confirmed MIAT as biomarker for chagasic cardiomyopathy (Frade et al., 2016)(**Table 1**). Also, during *T. gondii* (PTG and RH strains) infection in myDD8 (wild type) and myDD8^-/-^ (knock out) mice macrophages, siva1–205 and nfkb1–210 exhibited greater expression in myDD8 than Myd88^-/-^ macrophages when infected with *T. gondii* RH (Menard et al., 2021). These lncRNAs can thus serve as biomarkers for toxoplasmosis in a strain-specific manner and with respect to infection of mice macrophage. So, specific lncRNAs could appear as disease determinants or important indicators of parasitic infection (Menard et al., 2021), and as such, can serve as loop for selectable markers in host for disease diagnoses.

The existence of lncRNAs in EVs also creates the possibility of exploring these molecules as biomarkers for diagnosing parasitic diseases. EVs that enclosed lncRNAs have shown the possibility of modulating the response of recipient cells to drugs through intercellular transfer of specific drug resistant lncARSR (Zhou and Chen, 2019). A similar report is yet unknown in parasitic infection. Nevertheless, the epigenomic regulations in parasites and hosts, continuous identification drug resistance genes (Cowell and Winzeler, 2019), lncRNA sorting in EV secretions (**Figure 3**), and translational products in hosts and parasites may soon culminate into identification of gene-conjugated lncRNAs that may serve as targets for therapeutic molecules and diagnosis. Such a breakthrough will enhance our understanding of gene expression patterns to optimize drug efficacy and diagnostic tools. Therefore, future works are encouraged to identify circulating lncRNAs as biomarkers and therapeutic targets during parasitic infections.

## Hindsight and Perspectives

Significantly, adopted methods for assembling lncRNA algorithms play important roles in lncRNA expression, identification, and biochemical activity (Shields et al., 2020). In addition, lncRNA annotation resources could have unequal sensitivity to transcript abundance, uniqueness, functional complementarity, integrative characterization (Xu et al., 2017), and genomic features (Oliveira et al., 2018). Absence of (or partial) sequenced genomes, transcript assembling tools, and incomplete gene annotations are constraints to lncRNA annotation (Liao et al., 2018; Zheng et al., 2021). Continuous improvement on data assembling algorithms would enhance our capacity to detect new lncRNAs and their coding potentials (Hassan et al., 2012). However, identification of protein coding tendencies in time and space are still challenging. Also, the emergence of new putative linRNAs from existing genome annotation is still unclear. Would such phenomenon be due to algorithmic impasses, spatio-temporal gene switching or alternative transcript splicing? Similarly, there are growing studies on qRT-PCR analyses for lncRNA regulations, but qRT-PCR up/down regulations or bioinformatics predictions lack clinical interpretation, and the choice of lncRNAs for qRT-PCR are sometimes subjective or may not correlate with the result of RNA sequence (Vasconcelos et al., 2017).

In parasitic disease, several roles of lncRNAs in apoptosis, cellular differentiation/response (Fan et al., 2020), parasite biology, therapeutic targets, and drug resistance (Oliveira et al., 2018) are still inconclusive (Akay et al., 2019; Menard et al., 2021) (**Table 1**) (**Figure 2**). RNA immunoprecipitation would be useful in determining lncRNA functions during gene regulation in direct association with chromatin and epigenetic control of virulence (Sierra-miranda et al., 2012; **Table 1**). Similarly, genome editing, RNA binding assays, and gene knockdown would reveal the regulatory role of lncRNAs (Broadbent et al., 2011; Broadbent et al., 2015). When and if applicable, the use of RNAi and genome editing may show an incompletely captured subtle phenotype mediated by lncRNAs. Also, an epitranscriptomic approach may uncover novel lncRNA and peptide translation (Kim et al., 2020). Though, individual or group deletion of lncRNA genes may have different (un)discernable phenotypes and getting to know which set of lncRNAs present a particular trait may also be puzzling (Akay et al., 2019).

Parasites, more often than not, are distantly related. Consonant with this, lncRNAs with 100% sequence similarity are likely to function in parasite-specific or host-specific mode. As well, lncRNA domains that are pertinent to its structures may be deciphered through the primary sequence but may not give a determinate range of its function in conjunction with other molecules. Also, lncRNA inherent features of regulatory plasticity are of considerable concern for experimental designs (Li et al., 2020) especially the dual role of simultaneous gene activation and suppression (**Figure 2**). The understanding of ‘when’ and ‘how’ such parallel functions come to play is germane for future studies. And by extension, most long non-coding transcripts have no known function yet (Menard et al., 2021).

The involvement of lncRNAs in gene regulation potentially makes them important trade tools in the search for new therapeutics or biomarkers for many diseases (Rochet et al., 2019). LncRNAs generally have low primary sequence conservation which is more likely to distinguish specific parasite and/or strain infection than can be inferred with protein-coding genes. Also, lncRNAs that are intertwined with chromatin markers could be selected for functional analysis in order to understand their function in such loci relative to protein coding genes whilst lncRNAs with definite patterns in the infective stages of parasites may be good selections for studying host cell invasion and sexual development (Amaral et al., 2020). Considerations may equally be given to differentially expressed lncRNAs after drug treatment to identify functions of lncRNAs in parasite drug resistance and susceptibility.

During parasite development and survival in hosts, there are offsetting processes against parasite invasion through the expression of immune-related lncRNAs, some of which can be beneficial or detrimental to host and/or parasites. Then, what are the factors that ‘pre-program’ lncRNA activation for beneficial/detrimental traits during parasite infection in host or developmental changes of parasite? It has been proposed that several lncRNAs could regulate a gene and several genes could be regulated by a single lncRNA (Xia et al., 2020) for definite phenotype. In addition, variations in amino acid sequence ensue antigenic variation for which lncRNAs are involved *via* gene activation, protein binding, and chromatin conformational changes (Amit-Avraham et al., 2015). Since some lncRNAs can code for small peptides, it is still unknown if lncRNAs confer selective pressures on DNA/mRNA and/or encode antigenic peptides.

Identifying parasite exosomal lncRNAs and their export pathways would clear the coast further on the complex host immune network of action (Dragomir et al., 2018) during host-parasite interface. LncRNAs are active regulatory elements for retrograde takeover of host cells and immune escape for parasites but mechanistic designs for lncRNA in immune-related functions are still sparse (Zhang and Cao, 2016; Rochet et al., 2019). To this end, lncRNA functional analyses, in parasitic infections, should be prioritized and guided in pertinence to parasite biology and clinical relevance.

## Conclusion

The functional versatility of lncRNAs relies on their flexible conformational structures and wide-ranging tendencies to interact with diverse molecules. While certain lncRNAs exert their functions through interactions with hetero-nuclear chromatin complexes, others alter the stability or translation of mRNA in the cytoplasm. More importantly, lncRNA abundance, diversity, and dynamic expression across parasite stages set them as a potential one-stop-search to understand diverse processes in parasite development, host-parasite interactions, transcriptional regulation, and specific expression for determinate (genetic and phenotypic) traits. LncRNAs are activators/suppressors of host immune regulatory cascades and could be important tools for diagnosing parasitic diseases. Although there are existing gaps in our understanding of lncRNA functional threshold in parasitic infections, especially in helminths, it is in no doubt that these RNA molecules are paving the way for better understanding of parasite development and parasite-host crosstalk *via* modulation and fine-tuning of gene elements, as well as supervision of complex molecular interactions.

## Author Contributions

JC proposed the theme and provided guidance. JO organized the paper frame and drafted the manuscript. BO read the manuscript. All authors contributed to the article and approved the submitted version.

## Funding

Key Technologies Research and Development, R&D program, 2017YFD0500403JC.

## Conflict of Interest

The authors declare that the research was conducted in the absence of any commercial or financial relationships that could be construed as a potential conflict of interest.

## Publisher’s Note

All claims expressed in this article are solely those of the authors and do not necessarily represent those of their affiliated organizations, or those of the publisher, the editors and the reviewers. Any product that may be evaluated in this article, or claim that may be made by its manufacturer, is not guaranteed or endorsed by the publisher.
